# Distinctive approach in brain tumor detection and feature extraction using biologically inspired DWT method and SVM

**DOI:** 10.1038/s41598-023-50073-9

**Published:** 2023-12-20

**Authors:** Ankit Kumar, Saroj Kumar Pandey, Neeraj varshney, Kamred Udham Singh, Teekam Singh, Mohd Asif Shah

**Affiliations:** 1 Department of Information Technology, Guru Ghasidas Vishwavidyalaya, Bilaspur, India; 2 Department of Computer Engineering & Applications, GLA University, Mathura, Uttar Pradesh India; 3 School of Computer Science and Engineering, Graphic Hill Era University, Dehradun, 248002 India; 4 Department of Computer Science and Engineering, Graphic Era Deemed to be University, Dehradun, 248002 India; 5 Kebri Dehar University, Kebri Dehar, Somali 250 Ethiopia; 6 Centre of Research Impact and Outcome, Chitkara University Institute of Engineering and Technology, Chitkara University, Rajpura, 140401 Punjab India; 7 Division of Research and Development, Lovely Professional University, Phagwara, Punjab 144001 India

**Keywords:** Cancer, Computational biology and bioinformatics, Engineering

## Abstract

Brain tumors result from uncontrolled cell growth, potentially leading to fatal consequences if left untreated. While significant efforts have been made with some promising results, the segmentation and classification of brain tumors remain challenging due to their diverse locations, shapes, and sizes. In this study, we employ a combination of Discrete Wavelet Transform (DWT) and Principal Component Analysis (PCA) to enhance performance and streamline the medical image segmentation process. Proposed method using Otsu's segmentation method followed by PCA to identify the most informative features. Leveraging the grey-level co-occurrence matrix, we extract numerous valuable texture features. Subsequently, we apply a Support Vector Machine (SVM) with various kernels for classification. We evaluate the proposed method's performance using metrics such as accuracy, sensitivity, specificity, and the Dice Similarity Index coefficient. The experimental results validate the effectiveness of our approach, with recall rates of 86.9%, precision of 95.2%, F-measure of 90.9%, and overall accuracy. Simulation of the results shows improvements in both quality and accuracy compared to existing techniques. In results section, experimental Dice Similarity Index coefficient of 0.82 indicates a strong overlap between the machine-extracted tumor region and the manually delineated tumor region.

## Introduction

Recently, a significant medical Repeated Word science and imaging effort focused on tumor identification. Tumor detection remains a hugely tricky area of research in medical repeat word science. Our primary goal is to detect a brain tumor using proper and accurate techniques to treat a human being properly. We use MRI images to identify the tumor as an input image source. A brain tumor is an abnormal growth of brain cells and tissues^1^. A brain tumor can strike anyone at any age. The symptom of a brain tumor is determined by its size, type, and location. Vomiting, headaches, vision problems, and mental changes are all possible symptoms. Despite other symptoms, you may have difficulty walking and transitions in your speaking, hearing, and memory. Brain tumor detection and feature extraction are important areas of research in medical imaging. Detecting brain tumors early can significantly improve the chances of successful treatment, and extracting meaningful features from medical images can help doctors make more accurate diagnoses. Several techniques can be used for brain tumor detection and feature extraction, including:

- **Magnetic Resonance Imaging (MRI**): MRI is a non-invasive imaging technique that uses strong magnetic fields and radio waves to produce detailed images of the brain. MRI is often used for brain tumor detection and can also be used for feature extraction ^2^.
- **Computed Tomography (CT):** CT scans use X-rays to produce detailed images of the brain. CT scans can be used for brain tumor detection and feature extraction.
- **Biologically inspired methods:** Biologically inspired methods, such as the Discrete Wavelet Transform (DWT) and the Gabor transform, can be used for feature extraction in medical images. These methods are based on models of the human visual system and can be used to extract features related to texture, shape, and intensity^3^.
- **Machine learning:** Machine learning algorithms, such as Support Vector Machines (SVM) and Artificial Neural Networks (ANN), can be used for brain tumor detection and feature extraction. These algorithms can learn to classify images based on the extracted features.

The choice of technique will depend on several factors, including the availability of imaging equipment, the type of tumor being detected, and the specific research goals. Overall, brain tumor detection and feature extraction are important areas of research that can have a significant impact on patient outcomes.

The American Brain Tumor Association reports that nearly 80,000 men, women, and children were diagnosed with brain tumors in 2017^3,4^. In this particular scenario, wavelet transformation is used for digital pictures to extract features from such images and minimize the size of those features. The next step that we took was to use principal component analysis. We used a support vector machine to divide medical photos into either of the two groups. The kernel support vector machine was used in this study. Too many nouns (KSVM) with four kernels are used for accurate measurements. The kernel function is a quadratic function that can be radial, linear, or polygonal. Standard deviation, energy, mean, variance, Entropy, RMS, IDM, kurtosis, contrast, smoothness, correlation, and skewness are all calculated. Brain tumor detection is an important area of research in medical imaging, as early detection and diagnosis of brain tumors can significantly improve the chances of successful treatment. Biologically inspired wavelet transform methods, such as the Discrete Wavelet Transform (DWT), be effective for feature extraction in medical images. The DWT is a popular signal processing technique that decomposes signals into different frequency bands using wavelet functions. This decomposition can be used to extract features from medical images, such as texture and shape, which can then be used for classification purposes. A machine learning algorithm, Support Vector Machines (SVM) is useful for both classification and regression purposes. To perform its task, SVM searches the feature space for the hyperplane that creates the largest gap between the classes^5^.

Many imaging modalities, such as Positron Emission Tomography (PET), Computer Tomography (CT), and magnetic resonance imaging (MRI), can be used to identify brain cancers in humans. MRI remains popular because it provides high-density pixel values of the internal structure of the brain as well as helpful information for medical diagnosis and biomedical research. Image features (essential data patterns) such as image color information, texture, and shape aid in recognizing an image for the user query while searching and browsing image data. This proposed study will explore the database using medical images. The colors and textures of most image data are similar^6^. As a result, edge detection can assist in locating a more appropriate image in the database. Thus, edge detection techniques are investigated in this study to develop a new and improved methodology.

Major Objective

1. The main objective of the research is to detect brain tumors in medical images, specifically utilizing the advantages of Digital Wavelet Transformation (DWT) and Principal Component Analysis (PCA) for feature extraction and classification. This contributes to the critical field of early tumor detection in medical imaging.
2. The research leverages DWT and PCA for feature extraction from digital medical images. DWT is employed to reduce the size of extracted features from the images, which can be particularly important for large medical datasets.
3. The research uses a Support Vector Machine (SVM) for classifying medical images into two groups, namely those with and without brain tumors. This demonstrates the application of machine learning in medical image analysis.
4. The study utilizes a Kernel Support Vector Machine with four different kernels, including radial, linear, and polynomial functions. The choice of kernels allows for testing the performance of different classification techniques.
5. Various feature metrics are calculated, including standard deviation, energy, mean, variance, entropy, root mean square (RMS), inverse difference moment (IDM), kurtosis, contrast, smoothness, correlation, and skewness. These metrics provide valuable information for image classification and tumor detection.
6. The study explores image color information, texture, and shape as essential data patterns for image recognition in medical databases. Edge detection techniques are investigated to enhance image retrieval and recognition within the database.

### Organization of paper

The paper is organized down into several major sections, including an introduction that emphasizes the importance of brain tumor detection, a detailed explanation of the methodology involving DWT and PCA-based feature extraction, the application of SVM with various kernels for classification, and a discussion of feature metrics. The various related works have been discussed in section “Related work”. The paper concludes by stressing the biomedical significance of the research conducted, and it does so by discussing the importance of various medical imaging modalities, most notably MRI.

## Related work

In this section, we have reviewed the various existing reviews and discussed their method and result to identify the research gap. According to most research, many people have died due to the false detection of brain tumors. The proposed work is inspired by an article^2^ stating that noise-free MR images are fed into the k-means Algorithm to extract tumors. The Fuzzy C Means algorithm then calculates the feature extraction for accurate tumor extraction. Because it is well known that a brain tumor is a fatal disease that kills people. Tumors differ, and each tumor requires a unique treatment. There are many techniques for identifying tumors in the human body, such as CT scans, PET scans, MRIs, and so on, but when looking for malignancies in the brain, MRI is the most reliable method^3,7^. Our other task is to identify the selected features from the various elements. We find that PCA (Principal Component Analysis) is a common technique for finding high-dimensional patterns. It is a potent tool for data analysis.

It implied that the program would be intrigued by detective work broken tissue with a specific brightness intensity from its greyscale image. The broken tissues can be detected by thresholding. A series of filters will be used to eliminate noise, including mathematician, linear, and average filters^4^. Four modules, including preprocessing, segmentation, feature extraction, and classification, are recommended by^5^'s authors to aid in the detection of brain cancers. At the start of the procedure, the RGB to Grey Converter and median filter are used to preprocess the input MRI brain image. Preprocessing ensures that the image is ready for further processing before proceeding. Following this step, images are segmented using the histogram-based segmentation technique in the segmentation module. The segmented image can be used to derive many statistics, such as the mean and area, as well as measures of covariance and correlation, with the help of the feature extraction module. A Neural Network was used to categorize brain tumors, and another Neural Network completed this classification. The authors of ^6^ proposed a technique that combines them as a powerful tool for distinguishing traditional and abnormal human brains and detecting GRB kernels due to the triple-crown one. This brain imaging classification system, which PCA and KSVM support, could be a valuable tool for laptop-aided clinical identification. Tumor diagnosis using fuzzy C is successful, according to the authors of^7^, which suggests that clump is being applied frequently to genetic formula parameters. It is divided into two stages: preprocessing and post-processing. This system is used on various neoplasm sizes and intensities, whether primary or secondary abnormal images.

According to the concept presented in^8^, using the grey-level co-occurrence matrix for astrocytoma brain identification could be a novel method for brain cancer classification (GLCM). She was looking for signs of brain tumors using Artificial Neural Networks’ Back-Propagation Networks (VPNs) and Probabilistic Neural Networks (PNN) (PNN). The authors of^9^ suggested that the grey-level distribution of the image pixels was not sufficiently distinct; thus, using a specific comprehensive threshold worth to segment the entire image, including the tumor itself, was not the best choice, and concluded that the global thresholding technique provides poor results when attempting to section MRI images of the tumor-bearing brain.

The experimental comparison of applied math quantized bar chart texture features for successful image retrieval, inexactness, and recall in the DCT domain supported median and Laplacian filters, according to^10^. This information is then used to calculate several applied math texture options, such as kurtosis (kurtosis), smoothness (smoothness), energy, skewness, mean, standard deviation, and entropy. For image retrieval, the quantized bar chart texture options supported the Laplacian filter with edge extraction, median, and median, providing the most specific performance.

Authors^11^ discovered that the GUI-based application was far superior to standard tumor detection methods. The graphical user interface-based applications allow us to change settings without rewriting the software and quickly and efficiently identify tumors. The findings are more accurate and faster. Researchers^12^ concluded that they could differentiate between healthy tissues of the brain, such as white matter and grey matter, as well as cerebrospinal fluid and tumor-infected tissues by using MR images of the brain. Brain tumor identification and classification based on the Berkeley wavelet transform, support vector machine (SVM) and Back Propagation is compared to the ANFIS, Back Propagation, and NN classifiers. Back Propagation is also compared to support vector machine (SVM). The analysis provides more significant results by upbound measures such as mean, MSE, and PSNR, as well as accuracy, sensitivity, specificity, and dice constantly. It is quicker and more accurate than radiologists' or clinical consultants' manual identification of tumors.^13^ Proposed interactive segmentation to quickly and accurately segment brain tumors in tomography. The tumor and its size are effectively extracted using MATLAB programming for image processing and segmenting distinct brain CT images. The tumor size varies slightly depending on the slice of brain image used to calculate it.

According to^14^, the system successfully detects bone cancer from MRI scan images. At the end of the system, the system achieves its desired prospect. The extracted image features contain specific information that can be used to understand the picture's details. The main goal of removing the characteristics is to reduce process complications and isolate many desired image shapes. The extracted features can help to improve the classification stage's accuracy.^15^ demonstrated a segmentation technique that enables users to quickly and efficiently separate tumors in brain MRI. For greater efficiency, the Otsu thresholding technique is used in this method. The results show that Watershed Segmentation can successfully segment a tumor in MATLAB if the parameters are set correctly. They're making use of digital image processing techniques.

The paper^15^ proposes a novel approach for brain tumor localization and segmentation from MRI images. This method employs a preprocessing technique that analyses a tiny region of the picture rather than the entire image to circumvent the complexity and slowness of conventional approaches. This reduces computational time and aids in fixing overfitting issues. The second part of the process is the authors' proposal of a straightforward and effective Cascade Convolutional Neural Network (C-CNN) model for mining local and global information using separate but complementary methods. To further enhance the accuracy of brain tumor segmentation over previous models, the authors present the innovative Distance-Wise Attention (DWA) method. The DWA mechanism accounts for the impact of the tumor’s and the brain's shared central placement inside the model. Evaluated on the BRATS 2018 dataset, the suggested method produces competitive results, with mean whole tumor, enhanced tumor, and tumor core dice scores of 0.9203, 0.9113, and 0.8726, respectively. Extensive tests and assessments, including quantitative and qualitative analyses, are included in the study to back up the claims made for the efficacy of the suggested strategy.

This research^16^ introduces a novel framework for autonomous text localization and segmentation using convolutional neural networks (CNNs) in real-world settings with complicated backdrops. The detection of texts in such environments can be challenging due to factors such as high angles, profiles, dimensions, and colors. The proposed framework addresses these challenges and achieves high efficiency in detecting text. With a new inception layer and an enhanced ReLU layer, the system achieves top-notch results when it comes to text detection on complicated backdrops. Using the new m.ReLU building block and inception layer, the authors can maximize the detection of critical information while still exploring low-level visual aspects. The authors additionally investigate the impact of stacked inception layers (kernels with the dimension of 3 3 or greater) and demonstrate that this approach is superior to a linear chain of convolution layers at identifying texts of widely variable sizes (Conv layers). The suggested text detection method is tested using the International Corpus of Digital Audio Research (ICDAR) 2013, 2015, 2017, and 2019 databases. The created technique exceeds state-of-the-art frameworks, as shown by the findings, which show that it has the highest recall (94.2%), accuracy (95.6%), and F-score (94.8%). The findings on all of the aforementioned databases show how well the proposed system performs in identifying text against complicated backdrops in natural settings.

The present study^17^ highlights the significance of early detection of brain tumors and the challenges involved in achieving it. In this article, we take a close look at how artificial intelligence may be used to analyze MRI scans for signs of brain tumors, with a focus on Supervised, Unsupervised, and Deep Learning (DL) methods. MRI is a noninvasive imaging technique used for diagnosing and segmenting brain tumors. The risk of incorrect diagnoses has grown, however, because the number of doctors who are educated to use new technologies has lagged. Hence, research into methods of automating the identification of brain tumors has emerged as an important topic of study. The research summarizes the state-of-the-art in artificial intelligence (AI) brain tumor detection technologies and gives an appraisal of their efficacy. The publication also addresses unanswered questions and makes recommendations for further study. Overall, the study highlights the potential of AI methods in improving the early detection and diagnosis of brain tumors, which could significantly enhance the survival rates and quality of life of patients.

The article^18^ delves into LeNet-5’s architecture for image processing, object detection, and classification through the Shuffled Frog-Leaping Algorithm (SFLA). CNN has been widely adopted in the machine learning and computer vision communities thanks to its weight-sharing feature and efficient processing of information that is both spatially and temporally dispersed. Despite this challenge, a variety of optimization techniques have been employed to fine-tune the weights and biases of CNNs during training. Some examples of these methods are simulated annealing, genetic algorithms, ant colony optimization, and harmony search. In this paper, we use SFLA, an evolutionary trainer inspired by nature, to train the LeNet-5 architecture and compare the results to those obtained with other evolutionary trainers like the Whale Optimization Algorithm (WO), the Bacteria Swarm Foraging Optimization Algorithm (BFSO), and the Ant Colony Optimization Algorithm (ACO) (ACO). The study examines the SFLA algorithm’s performance across four datasets, finding that it significantly improves the performance of the original LeNet-5 architecture, albeit slightly increasing the training computation time. High classification and approximation accuracy in the algorithm’s mechanism is also shown by the findings.

In this study^19^, we offer an autonomous approach to breast tumor segmentation and detection using a shallow convolutional neural network (CNN) with many paths for extracting features. The goal is to speed up the process of identifying breast cancers without sacrificing precision. By applying the photographs to the model, the suggested method applies an image enhancement strategy to boost their quality. This approach avoids the need for a very deep structure in the CNN, which can be computationally expensive. The study evaluates the proposed method on two datasets, Mini-MIAS and the Digital Database for Screening Mammography (DDSM), and provides excellent tumor localization and classification accuracy. For normal, benign, and malignant areas, the model obtains an accuracy of 0.936, 0.890, and 0.871 on the DDSM dataset and 0.944, 0.915, and 0.892 on the Mini-MIAS dataset. The proposed method provides a significant improvement in detecting the borders of tumors, which is a critical aspect of tumor segmentation. The use of multi-feature extraction routes in the shallow CNN allows for the extraction of important features that represent the boundaries of tumors. This addresses a significant challenge in breast cancer diagnosis, where benign and malignant tumors can be difficult to distinguish if the borders are not segmented correctly.

A new CNN architecture is proposed in this study^20^ that integrates data from four picture modalities to enhance segmentation precision. Each modality's features are extracted using a ResNet-50 network in the proposed model, and additional features are extracted using two basic building components. By establishing patches of two sizes, we may integrate local and global characteristics. The model is trained and evaluated on the BRATS 2018 dataset, which is a widely used benchmark for brain tumor segmentation. The results show that the proposed architecture achieves competitive dice scores for the enhanced, whole, and core tumor areas. The use of multiple modalities and the incorporation of ResNet-50 weights and biases are innovative features of this study. The proposed architecture takes advantage of both local and global features, which can enhance the accuracy of tumor segmentation. The results demonstrate the potential of the proposed approach for clinical use in the diagnosis and treatment of brain tumors.

This study^21^ presents an AI-based approach for automatic brain tumor detection and segmentation using MRI images. The authors utilized a convolutional neural network (CNN) model to classify and segment brain tumors from MRI images. The study achieved high accuracy in tumor detection and segmentation, and the authors suggest that their method could assist radiologists in clinical practice.

In this study^22^, the authors developed a deep learning model to predict the molecular subtype of brain tumors using MRI images. The authors utilized a multi-scale 3D CNN model to extract features from MRI images and then used these features to predict the molecular subtype of the tumor. The study achieved high accuracy in molecular subtype prediction, and the authors suggest that their model could aid in personalized treatment planning for brain tumor patients.

This study^23^ proposes a hybrid model for brain tumor detection and segmentation using MRI images. The authors combined a CNN model with a random forest classifier to improve the accuracy of tumor detection and segmentation. The study achieved high accuracy in tumor detection and segmentation, and the authors suggest that their hybrid model could be used as a clinical decision-support tool for brain tumor diagnosis and treatment planning.

Five distinct animals (mice, rats, grey short-tailed opossums, white sturgeons, and great horned owls) were imaged with a custom-built SS-OCT system to collect 3D retinal and vascular OCTA data^24^. Scientists were able to create detailed enface visualizations of the retina and choroidal vascular plexus, as well as high-resolution 2D photographs of the retina and choroidal components. The study discovered that the rat and mouse retinas had comparable morphologies and vascular plexuses, whereas the opossum retina had clear choroidal and paired superficial arteries. Pecten oculi and the underlying avascular and choroidal vasculature in the owl retina were likewise recorded, as were their respective locations and functions, as were the sturgeon's retina and vascular anatomy.

This investigation^25^ focused on the directional reflectivity of the retinal layers, paying special attention to scattering from the retinal pigment epithelium (RPE). The retinal pigment epithelium (RPE) of three mouse strains was compared: albino (BALB/c), agouti (129S1/SvlmJ), and dark-skinned (C57BL/6 J). Using a directed OCT device, the researchers shifted the location of the narrow OCT beam's entrance pupil as it traveled across the dilated pupil of the mice's eyes to assess the strength of the backscattering signal. The internal and exterior limiting membranes, as well as Bruch's membrane, were also analyzed and compared amongst the different strains of mice (in albino mice). Although the IS/OS junctions were found to be mostly unaffected by illumination, it was discovered that the intensity of light backscattered from the melanin-free layers depended significantly on the angle at which it was incident. Retinal pigment epithelium reflections were somewhat ordinary in animals with high melanin levels. From strain-to-strain differences in directional scattering, it seems that increasing levels of melanin in the RPE lead to less contribution from Mie scattering by melanosomes.

In this study^26^, we provide a novel automated calibration technique for swept-source optical coherence tomography (SS-OCT) devices. The suggested technology enables real-time calibration during scanning without requiring a dedicated calibration interferometer or a separate channel. Rather, the reflection from the sample's surface is employed as the calibration signal's spectral component. Using this signal, we can calculate the phase function that represents the frequency-swept laser source’s non-linear sweeping characteristic, and we can then normalize and rescale the phase to make it linear. To resample the OCT signals, fractional-time indices are derived directly from the resampling function, allowing for perfect calibration despite non-planar topography or galvo scanning. Using an in-house built SS-OCT system, the suggested technique was proved to enable high-performance calibration without adding computational or hardware complexity. When applied to complicated imaging scenarios, like microsurgery, this innovative automated calibration approach greatly enhances the precision and efficiency of SS-OCT imaging.

In this research^27^, we provide a new approach to time-domain resampling for swept-source optical coherence tomography (SS-OCT) data. To get at the raw phase values included in a Mach–Zehnder interferometer calibration signal, the suggested approach employs phase analysis. Once the phase values are calculated, they are normalized and scaled to create uniform wavenumber spaces. In contrast to previous phase-based time domain resampling techniques, ours directly computes the non-uniform fractional time index values corresponding to the uniformly distributed phase values from the linearizer coordinates. The suggested method outperforms previous approaches in terms of both accuracy and speed. In-house SS-OCT system images of a human fingernail and an eye model are used to show the algorithm's resilience and influence on resolution and sensitivity.

Gastrointestinal (GI) diseases pose a considerable obstacle to healthcare institutions, necessitating the utilisation of sophisticated detection techniques^48^. Although deep neural networks (DNNs) have demonstrated exceptional performance in a range of imaging tasks, their potential for detecting gastrointestinal (GI) diseases has not been extensively investigated. This study presents a novel methodology for the identification of gastrointestinal disorders using wireless capsule endoscopy (WCE) images, employing Convolutional Neural Networks (CNNs). The proposed approach encompasses the utilisation of three modified backbone networks, which have been further enhanced through the application of transfer learning techniques, in order to extract relevant features. Moreover, a classifier that utilises ensemble learning is trained to accurately detect and classify gastrointestinal diseases. The computational approaches currently available are outperformed by our method when applied to benchmark datasets. Furthermore, the results obtained from case studies provide further evidence of our method's capability to effectively capture discriminative information in wireless capsule endoscopy (WCE) images. This study highlights the capacity of employing deep learning-based computer vision models for efficient gastrointestinal disease screening.

Skin disorders are commonly found among individuals, and the utilisation of deep learning techniques has the potential to improve the precision of identifying these conditions. The uneven distribution of background information in dermatological datasets presents a significant obstacle for deep learning models that aim to achieve generalisation, thereby affecting their overall performance. In this study, we present a novel deep learning framework that integrates data preprocessing, data augmentation, and residual networks. objective of this paper^49^ to investigate the influence of color-based background selection on the ability of deep models to effectively learn attributes of foreground lesions in the classification of skin diseases. This methodology entails the process of dermatologists annotating the data and subsequently masking the original background information using different colours, resulting in the creation of subsets with varying background colours. Through the independent training of deep learning networks on these subsets, our study showcases the substantial impact of color-based background information on the classification of skin diseases.

The presence of foot and ankle deformities in paediatric patients can result in enduring consequences, underscoring the significance of timely identification and diagnosis. Nevertheless, the current diagnostic procedures exhibit a deficiency in terms of objectivity. The objective of this study is to develop a comprehensive assessment framework for paediatric foot and ankle deformities using techniques of data mining and machine learning. In this study, we present a set of grading rules for assessing the severity of deformities. Additionally, Author^50^ utilise a 3D foot scanner to gather data on 30-foot structure indexes. In this study, we present a novel sparse multi-objective evolutionary algorithm designed for the purpose of feature selection. Through empirical evaluation, we provide evidence of its efficacy and efficiency in conducting search operations. The algorithm under consideration streamlines the evaluation model, resulting in an average classification accuracy exceeding 98% through the utilisation of random forests.

This paper^51^ presents the Colony Predation Algorithm (CPA), a stochastic optimisation technique that draws inspiration from animal hunting strategies. The CPA framework employs mathematical mappings that simulate animal hunting strategies, including prey dispersion, encirclement, support for the most proficient hunter, and target switching. Furthermore, the Certified Public Accountant (CPA) presents a distinctive mathematical framework that incorporates success rates in order to adjust strategies and simulate the behaviour of selectively abandoning certain actions. The algorithm efficiently tackles cross-border scenarios by optimising the values of positions. Numerous empirical investigations have consistently shown that the Competitive Performance Algorithm (CPA) outperforms state-of-the-art metaheuristics across a range of search landscapes.

The health of patients is significantly affected by Osteoporotic Vertebral Fracture (OVFs), and the utilisation of CT images provides distinct demarcations of bone blocks, thereby assisting in the process of diagnosis. X-rays, despite their expedience and cost-efficiency, frequently result in misdiagnosis as a consequence of the presence of overlapping shadows. In order to extend the benefits of CT imaging to the classification of X-ray-based osteoporotic vertebral fractures (OVFs), we put forth a multi-modal semantic consistency network. The network utilises feature-level mix-up modules to mitigate domain discrepancies, incorporate domain soft labels, and employ self-rotation pretext tasks to improve the learning of high-level semantic invariant features. The method employed in study^51^ demonstrates superior performance compared to existing approaches, resulting in a notable enhancement of the Area Under the Curve (AUC) metric from 86.32 to 92.16%. This study showcases the efficacy of employing multi-modal semantic consistency in the classification of osteoporotic vertebral fractures (OVFs) using CT imaging features in X-rays shown in Table 1.

**Table 1:** Contains the comparative study of existing work to highlight the exciting work.

Sr. No.	Author	Year	Machine learning algorithms and accuracy (%)
1	Agravat and Raval^1^	2021	Brain tumor measure of size, shape, location, and a high volume of MR images, SVM: 63.5%, LR: 64.0, C4.5: 63%, KNN:55.15%
2	Alhassan et al.^2^	2021	Deep neural network 98.6% Accuracy
3	Shayari et al.^3^	2021	Bayesian optimization 97.37% Accuracy
4	Ayadi et al.^4^	2021	CNN network with 98% Accuracy
5	Barzegar and Jamzad^5^	2021	BRATS datasets including high- and low-grade glioma brain tumors, KNN: 86.32%, Naïve Bays: 60.46%,
6	Bashir-Gonbadi and Khotan Lou^6^	2021	IXI and BraTS 2017 datasets, used for proposed work and to classify Brain Tumor Naïve Bays: 86%
7	Jena et al.^7^	2022	Classification accuracy of 94.25%, 87.88%, 89.57%, 96.99%, and 97% with SVM, KNN, BDT, RF
8	Jiang et al.^8^	2021	Edge extraction algorithm for providing edge labels with Accuracy: 96.55%
9	Kadry et al.^9^	2021	Modified Moth-Flame Optimization algorithm with 83.4%
10	Kokkalla et al.^10^	2021	ResNet v2 with a deep, dense network and a soft-max layer with an accuracy of 99.69%
11	Qiaosen et al.^48^	2022	Automated detection of gastrointestinal diseases using wireless capsule endoscopy (WCE) images with Convolutional Neural Networks (CNNs) with accuracy rate of 94.8%
12	Jiancun et al.^49^	2022	Enhance the accuracy of skin disorder identification by studying the impact of color-based background selection on deep learning models' capacity to learn attributes of foreground lesions in skin disease classification with accuracy
13	Xiaotian et al.^50^	2022	Develop a model for children’s foot & ankle deformity using data mining and machine learning, including grading rules for deformity severity and 3D foot structure index data and achieve the accuracy of 98%
14	Jiaze et al.^51^	2021	Introduce a stochastic optimizer inspired by animal hunting strategies to improve optimization performance in various search landscapes. The experimental results show reveal that CPA’s average is about 0.9 lower than the second DE and about 0.84 lower than the second improved DECLS
15	Yuzhao et al.^52^	2022	Develop a multi-modal semantic consistency network for improved osteoporotic vertebral fracture classification in X-rays by utilizing CT imaging features and enhancing domain consistency. Proposed method improves the best value of AUC from 86.32 to 92.16%

## Proposed solution

This section includes a detailed description of the problem domain and a proposed solution. In addition to that, the detailed description and functional model are also listed in this section.

### Domain description

Image processing is the study of mathematical models that are used to improve image and image quality^28^. These methods are essential in real-world applications such as object recognition, information extraction, and search engines. To provide efficient and effective image retrieval, various image retrieval and extraction models are available^29^. In this context, shape, texture, and color analysis help separate images from massive databases based on the images queried. As a result, the current study aims to find the best method for calculating features in a medical database. Most image data with similar color and texture features are available in the medical domain^30^. As a result, the shape features to aid in image differentiation in this domain.

### Problem domain

The medical domain and image search face critical issues that must be addressed in the proposed work. The implementation of the desired technique, the performance and classification of SVM^31^ with the various kernel functions implemented in the system are evaluated, and the results are summarized. The brain tumor dataset was gathered at the Institute of Oncology, University Medical Centre Ljubljana, Yugoslavia. This section describes the proposed study's problem domain. The following section focused on the presented work's solution domain.

### Algorithms study

This section includes the algorithms utilized in the implementation of the presented Algorithm.

#### Otsu’s method

In image processing, the Nobuyuki Otsu method, named after a Japanese scientist, is well-known for automatically converting grey-level images to binary images. This system assumes that the image has two different pixels by using the bi-modal bar chart foreground and background pixels. Because intra-class variance is reduced, inter-class conflict is highest. As a result, Otsu's method is essentially a one-dimensional, isolated version of Fisher's Discriminant Analysis^32,33^.

When it comes to automated global thresholding, the Otsu approach^1^ is among the most well-known options out there. The pixels in the provided image can be represented by the L grayscale $\left[ {0,1,2, \ldots L - 1} \right]$. The total number of pixels in the image is $N = n_{0} + n_{1} + \cdots + n_{L - 1}$. Where n is the number of pixels in the current grayscale level (L−1). The histogram of grayscale values is scaled according to this probability distribution function.

1 $$p_{i} = \frac{ni}{N},p_{i} \ge 0,\mathop \sum \limits_{i = 0}^{L - 1} p_{i} = 1$$

We chose k as the cutoff value; the normalized proportion of pixels will be used to determine what constitutes the image’s foreground and background.

2 $$\begin{gathered} {\text{s}}_{{\text{x}}} \left( {\text{k}} \right) = \mathop \sum \limits_{{\text{i = 0}}}^{{\text{k}}} {\text{p}}\left( {\text{i}} \right) \hfill \\ {\text{s}}_{{\text{y}}} \left( {\text{k}} \right) = \mathop \sum \limits_{{{\text{i = K + }}1}}^{{{\text{L}} - 1}} {\text{p}}\left( {\text{i}} \right) \hfill \\ {\text{s}}_{{\text{x}}} \left( {\text{k}} \right) + {\text{s}}_{{\text{y}}} \left( {\text{k}} \right) = 1 \hfill \\ \end{gathered}$$

The average pixel gray value for the backdrop and the middle pixel gray value for an item will be:

3 $$\begin{aligned} \mu_{{\text{x}}} \left( {\text{k}} \right) & = \frac{{\mathop \sum \nolimits_{{{\text{i}} = 0}}^{{\text{k}}} {\text{i}}p\left( {\text{i}} \right)}}{{{\text{p}}\left( {\text{i}} \right)}} = \frac{1}{{s_{{\text{x}}} \left( {\text{k}} \right)}}\mathop \sum \limits_{{{\text{i}} = 0}}^{{\text{k}}} {\text{ip}}\left( {\text{i}} \right) \\ \mu_{{\text{y}}} \left( k \right) & = \frac{{\mathop \sum \nolimits_{i = k + 1}^{L - 1} ip\left( i \right)}}{p\left( i \right)} = \frac{1}{{s_{y} \left( k \right)}}\mathop \sum \limits_{i = k + 1}^{L - 1} ip\left( i \right) \\ \end{aligned}$$

The whole grayscale image, with its mean value determined by the Eq. (4)

4 $$\mu = \frac{{\mathop \sum \nolimits_{i = 0}^{L - 1} ip\left( i \right)}}{{\mathop \sum \nolimits_{i = 0}^{L - 1} p\left( i \right)}} = \mathop \sum \limits_{i = 0}^{L - 1} ip\left( i \right)$$

Variation in pixel gray value between the background and objects will be:

5 $$\begin{array}{*{20}c} {\sigma_{x}^{2} \left( k \right) = \frac{{\mathop \sum \nolimits_{i = 0}^{k} \left( {i - \mu_{x} } \right)^{2} p\left( i \right)}}{{\mathop \sum \nolimits_{i = 0}^{k} p\left( i \right)}} = \frac{1}{{s_{x} \left( k \right)}}\mathop \sum \limits_{i = 0}^{k} \left( {i - \mu_{x} } \right)^{2} p\left( i \right)} \\ {\sigma_{y}^{2} \left( k \right) = \frac{{\mathop \sum \nolimits_{i = k + 1}^{L - 1} \left( {i - \mu_{y} } \right)^{2} p\left( i \right)}}{{\mathop \sum \nolimits_{i = k + 1}^{L - 1} p\left( i \right)}} = \frac{1}{{s_{y} \left( k \right)}}\mathop \sum \limits_{i = k + 1}^{L - 1} \left( {i - \mu_{y} } \right)^{2} p\left( i \right)} \\ \end{array}$$

Then, the total grayscale image’s variance is computed by using the formula:

6 $$\sigma^{2} = \mathop \sum \limits_{i = 0}^{L - 1} (i - \mu )^{2} p\left( i \right)$$

Within-class and between-class variance the variance $\sigma^{2}$ can be defined as:

7 $$\begin{aligned} \sigma^{2} & = s_{x} \left( k \right)\sigma_{x}^{2} \left( k \right) + s_{y} \left( k \right)\sigma_{y}^{2} \left( k \right) + s_{x} \left( k \right)\left( {\mu_{x} \left( k \right) - \mu } \right)^{2} + s_{y} \left( k \right)\left( {\mu_{y} \left( k \right) - \mu } \right)^{2} \\ & = \sigma_{w}^{2} \left( k \right) + \sigma_{b}^{2} \left( k \right) \\ \end{aligned}$$

where $\sigma_{w}^{2} \left( k \right)$ is the within-class variance and $\sigma_{b}^{2} \left( k \right)$ is the between-class variance.

#### Wavelet transformation

Wavelet Transform: Wavelet transform can analyze an image by factoring it into a series of sub-band images. These sub-band images can be considered wavelet coefficients, where texture features are calculated. Any function can be regarded as a wavelet function Ѱ (t) if it satisfies the following conditions: A wavelet function's square integral is finite. It defines a wavelet must have a limited (small) energy.

8 $$\mathop \int \limits_{{ - \infty }}^{{ + \infty }} \left| {\varphi \left( t \right)} \right|^{ \wedge } 2dt < \infty$$

The integral of the wavelet function Ѱ (t) equals zero. It defines that the process must be oscillatory (wave).

9 $$\mathop \smallint \limits_{ - \infty }^{ + \infty } \varphi \left( t \right)dt = 0$$

#### Continuous wavelet transform (CWT)

CWT, computed wavelet coefficients at each achievable range and location, has made plenty of redundant in sequence and luxurious computing influences and moments in time. Transform^34^ becomes economical, efficient, and sensible in real-world applications to create wavelets.

#### Discrete wavelet transform (DWT)

**Figure 1:**
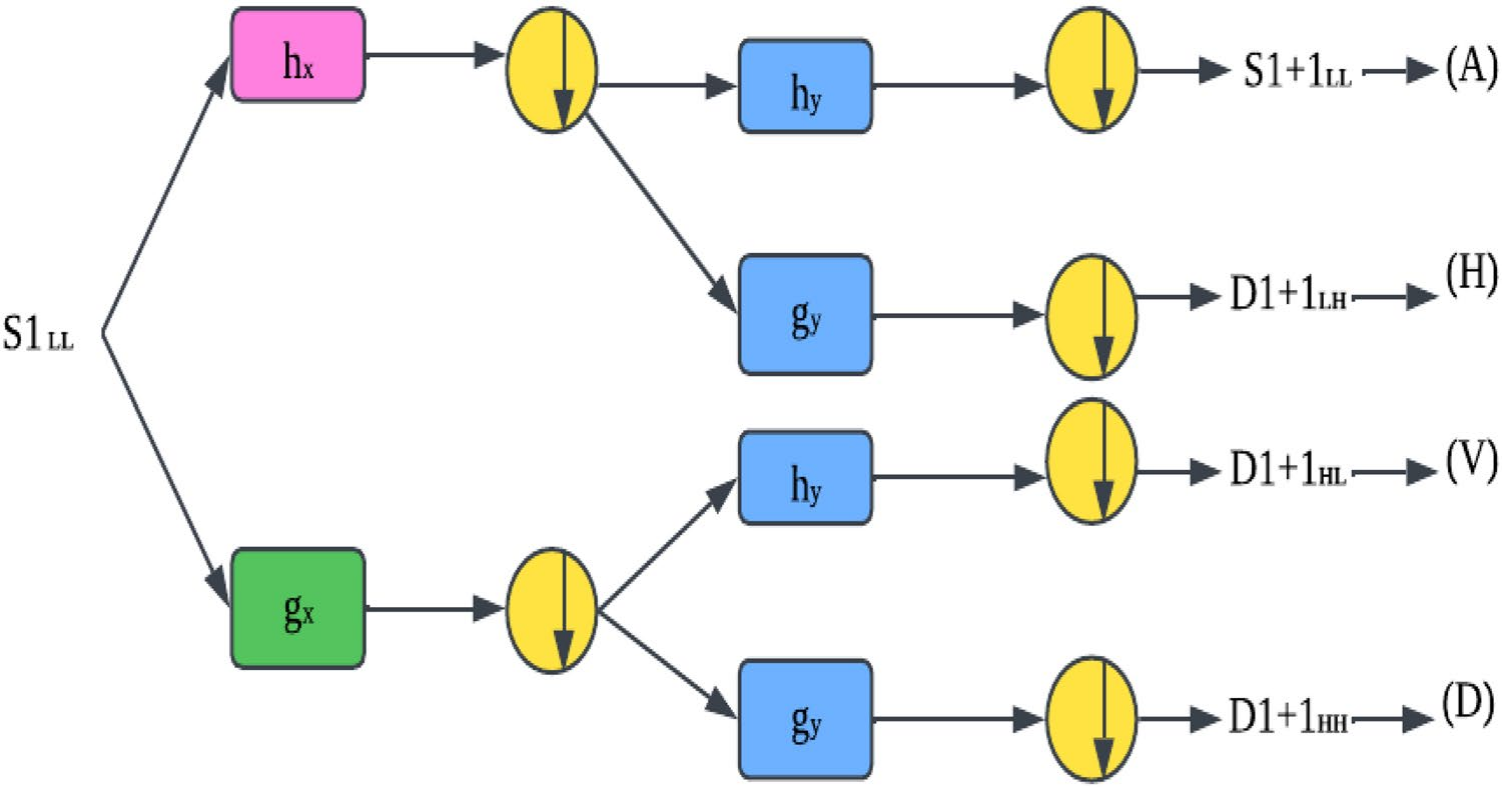
Data distributing in discrete wavelet transform.

DWT is accomplished by iteratively filtering an indication or likeness from beginning to end through the high-pass and low-pass filters and later sampling the filtered information by two. This procedure can decompose the input image into a series of subband pictures. Figure 1 illustrates the associated example of DWT, whereas the image with a down arrow within a circle represents the down-sampling operation.

**Figure 2:**
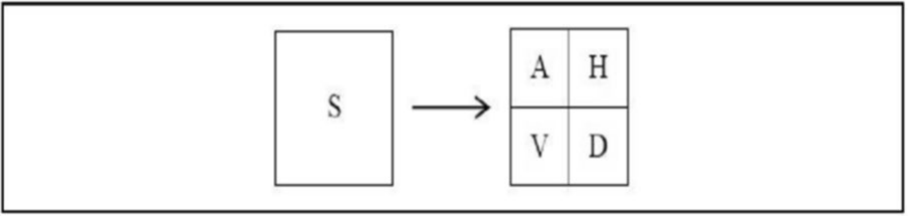
Sub-band images for one level of image decomposition using Discrete Wavelet Transform.

One stage of the decomposition method yielded four sub-band pictures from an image S at a resolution level. The low-frequency module of the original picture, S, is the approximation image. The output of a decomposed image using wavelet transform is shown in Fig. 2.

Every high-quality photograph may contain information of a specific direction and scale. This method also stores spatial information in the sub-band pictures. The following equations can be used to determine the sub-band images obtained from low-pass and high-pass filters:

10 $$\begin{aligned} A{ } & = { }\left[ {hx{ * }\left( {{ }hy{ * }S{ }} \right) \downarrow 2,1} \right]{ } \downarrow 1,2 \\ H{ } & = { }\left[ {hx{ * }\left( {{ }gy{ * }S{ }} \right) \downarrow 2,1} \right]{ } \downarrow 1,2 \\ V{ } & = { }\left[ {gx{ * }\left( {{ }hy{ * }S{ }} \right) \downarrow 2,1} \right]{ } \downarrow 1,2 \\ D{ } & = { }\left[ {gx{ * }\left( {{ }gy{ * }S{ }} \right) \downarrow 2,1} \right]{ } \downarrow 1,2 \\ \end{aligned}$$

S represents the original input image and the sampling operator.

Therefore, sampling is a procedure to withdraw those useless and filtered samples from the subband images. This approach is unsuitable for texture classification because texture features are translation-invariant.

#### Discrete wavelet frame transform (DWFT)

**Figure 3:**
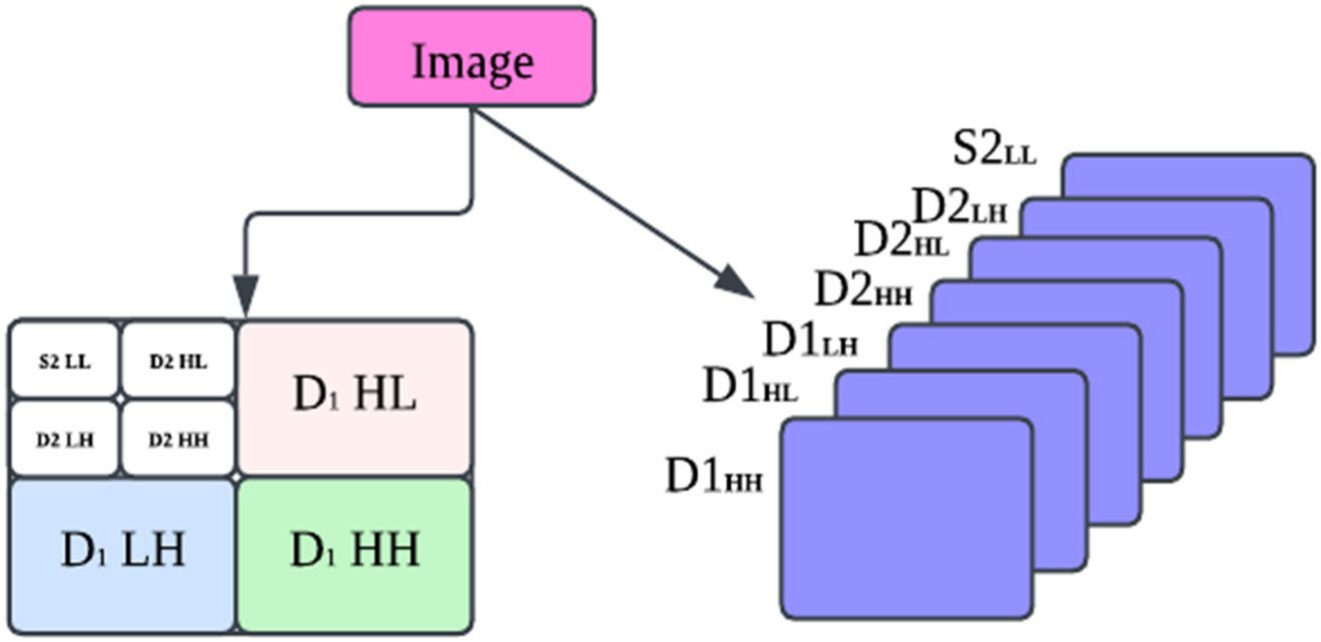
Show the difference between DWT and DWFT.

Some investigators have suggested the DWFT method in texture classification^35^. The difference between them is that DWFT does not implement the sampling operations and creates a translation-invariant property for the decomposition results, as shown in Fig. 3.

### Recognizes the pulse sequence and weighting

Recognizing the pulse sequence and weighting in the MRI images includes radiofrequency waves sent in short lines of pulses. Different arrangements provide different information about the tissues, differences between one series and another depending on the frequency pulses used and the time between them. The MR imaging examination is programmed as a series of pulse sequences in each region. There are two large families of lines.

- Spin echo sequence (SE for the initials in English).
- Gradient echo sequences (GRE, FFE).

The manipulation of the radiofrequency pulses and the times between them defines the enhancement of the image. The images are enhanced in T1, T2, and proton density (PD). The parameters that each one values are different. In MRI, the greyscale represents the intensity of the signal emitted by the tissue. Therefore, to describe the images, we refer to your signal. A tissue or structure is hyperintense when its coloration is white or light grey (it shines more intensely). On the other hand, if its coloration is dark (tendency to be black), it is hypointense^36^. The intermediate grey signal is described as intense.

#### Proposed algorithm

Algorithm 1: Proposed Algorithm for extracting segment image feature.
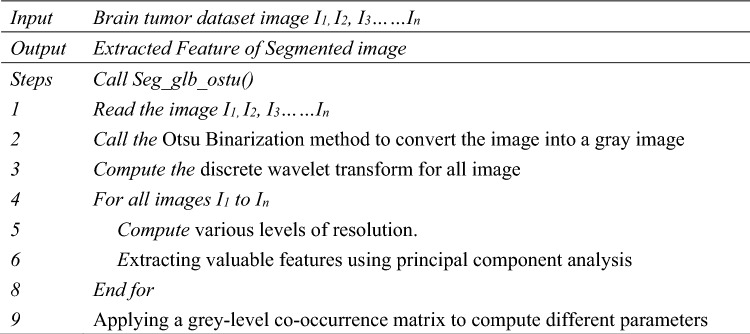


This is the revised goal function that takes into account both local and nonlocal factors:

11 $$\begin{aligned} J_{m} & = \mathop \sum \limits_{i = 1}^{c} \mathop \sum \limits_{k = 1}^{N} u_{ik}^{m} \mathop \sum \limits_{{r \in {\Omega }_{k} }} K\left( {r - k} \right)\left\| {x_{k} - b\left( r \right)v_{i} } \right\|^{2} \\ & \;\;\, + \mathop \sum \limits_{i = 1}^{c} \mathop \sum \limits_{k = 1}^{N} \left( {a_{k} u_{ik}^{m} \left( {1 - u_{ik}^{m - 1} } \right) + \beta_{k} u_{ik}^{m} \mathop \sum \limits_{{r \in {\Omega }_{k} }} K\left( {r - k} \right)\left\| {\overline{x}_{k} - b\left( r \right)v_{i} } \right\|^{2} } \right) \\ \end{aligned}$$

$u_{ik}$ Is the fuzzy clustering membership degree, $v_{i}$ is the ith cluster center, ${\Omega }_{k} { }$ represents a neighborhood window centered at $x_{k}$ with radius r, and $\overline{x}_{k}$ is the nonlocal computed value of the kth pixel. The definition of $K\left( {r - k} \right)$ and $b\left( r \right)$ is the same as for $u_{ik}$. $\beta_{k} { }$ It is a weighting parameter to balance local coupling items with nonlocal regularisation items.

12 $$\beta_{k} = \frac{{mk\left( {w_{k}^{u} } \right) - m{ }\left( {w_{k}^{u} } \right)}}{{m{ }\left( {w_{k}^{u} } \right)}}$$

Assume $\sum_{i = 1}^{c} u_{ik} = 1,0 \le u_{ik} \le 1$, and $m > 1$. The Lagrange multiplier approach may be used to calculate $u_{ik}$ and $v_{i}$ as^37^

13 $$\begin{aligned} u_{ik} & = \frac{{\mathop \sum \nolimits_{{r \in {\Omega }_{k} }} \left( {K\left\| {x_{k} - b_{k} v_{i} } \right\|^{2} - a_{k} + \beta_{k} K\left\| {\overline{x}_{k} - b_{k} v_{i} } \right\|^{2} } \right)^{{ - 1/\left( {m - 1} \right)}} }}{{\mathop \sum \nolimits_{l = 1}^{c} \left( {\mathop \sum \nolimits_{{r \in {\Omega }_{k} }} \left( {K\left\| {x_{k} - b_{k} v_{l} } \right\|^{2} - a_{k} + \beta_{k} K\left\| {\overline{x}_{k} - b_{k} v_{l} } \right\|^{2} } \right)} \right)^{{ - 1/\left( {m - 1} \right)}} ,}} \\ v_{i} & = \frac{{\mathop \sum \nolimits_{k = 1}^{N} \mathop \sum \nolimits_{{r \in {\Omega }_{k} }} Kb_{k} \left( {x_{k} + \beta_{k} \overline{x}_{k} } \right)u_{ik}^{m} }}{{\mathop \sum \nolimits_{k = 1}^{N} \mathop \sum \nolimits_{{r \in {\Omega }_{k} }} Kb_{k}^{2} \left( {1 + \beta_{k} } \right)u_{ik}^{m} }} \\ \end{aligned}$$

Unconstrained optimization may be solved using the Lagrange multiplier approach for

14 $$\begin{aligned} L & \left( {u_{ik} ,v_{i} ,} \right.\left. {b_{k} ,\lambda_{k} ,a_{k} ,\beta_{k} } \right) \\ & { = }\mathop \sum \limits_{i = 1}^{c} \mathop \sum \limits_{k = 1}^{N} u_{ik}^{m} \mathop \sum \limits_{{r \in {\Omega }_{k} }} K\left\| {x_{k} - b_{k} v_{i} } \right\|^{2} \\ { } & \;\;\; + \mathop \sum \limits_{i = 1}^{c} \mathop \sum \limits_{k = 1}^{N} \left( {a_{k} u_{ik}^{m} \left( {1 - u_{ik}^{m - 1} } \right) + \beta_{k} u_{ik}^{m} \mathop \sum \limits_{{r \in {\Omega }_{k} }} K\left\| {\overline{x}_{k} - b_{k} v_{i} } \right\|^{2} } \right) \\ & \;\;\; + \mathop \sum \limits_{k = 1}^{N} \lambda_{k} \left( {1 - \mathop \sum \limits_{i = 1}^{c} u_{ik} } \right) \\ \end{aligned}$$

Compute the partial derivative of $L$ concerning $u_{ij}$ and $\lambda_{k}$, respectively, and let $\partial L/\partial u_{ik} = 0$ and $\partial L/\partial \lambda_{k} = 0$; that is,

15 $$\begin{aligned} \frac{\partial L}{{\partial u_{ik} }} & = mu_{ik}^{m - 1} \mathop \sum \limits_{{r \in {\Omega }_{k} }} K\left\| {x_{k} - b_{k} v_{i} } \right\|^{2} + a_{k} - ma_{k} u_{ik}^{m - 1} \\ & \;\; + m\beta_{k} u_{ik}^{m - 1} \mathop \sum \limits_{{r \in {\Omega }_{k} }} K\left\| {\overline{x}_{k} - b_{k} v_{i} } \right\|^{2} - \lambda_{k} = 0 \\ \frac{\partial L}{{\partial \lambda_{k} }} & = 1 - \mathop \sum \limits_{i = 1}^{c} u_{ik} = 0 \\ \end{aligned}$$

From (8), we obtain

$$u_{ik} = \left( {\frac{{\lambda_{k} - a_{k} }}{{m\mathop \sum \nolimits_{{r \in {\Omega }_{k} }} \left( {K\left\| {x_{k} - b_{k} v_{i} } \right\|^{2} - a_{k} + \beta_{k} K\left\| {\overline{x}_{k} - b_{k} v_{i} } \right\|^{2} } \right)}}} \right)^{{1/\left( {m - 1} \right)}}$$

By substituting, we obtain

$$\left( {\frac{{\lambda_{k} - a_{k} }}{m}} \right)^{{ - 1/\left( {m - 1} \right)}} = \frac{1}{{\mathop \sum \nolimits_{l = 1}^{c} \left( {\mathop \sum \nolimits_{{r \in {\Omega }_{k} }} \left( {K\left\| {x_{k} - b_{k} v_{l} } \right\|^{2} - a_{k} + \beta_{k} K\left\| {\overline{x}_{k} - b_{k} v_{l} } \right\|^{2} } \right)} \right)^{{ - 1/\left( {m - 1} \right)}} }}$$

By substituting (8) with (9), we obtain^38^

17 $$u_{ik} = \frac{{\mathop \sum \nolimits_{{r \in {\Omega }_{k} }} \left( {K\left\| {x_{k} - b_{k} v_{i} } \right\|^{2} - a_{k} + \beta_{k} K\left\| {\overline{x}_{k} - b_{k} v_{i} } \right\|^{2} } \right)^{{ - 1/\left( {m - 1} \right)}} }}{{\mathop \sum \nolimits_{l = 1}^{c} \left( {\mathop \sum \nolimits_{{r \in {\Omega }_{k} }} \left( {K\left\| {x_{k} - b_{k} v_{l} } \right\|^{2} - a_{k} + \beta_{k} K\left\| {\overline{x}_{k} - b_{k} v_{l} } \right\|^{2} } \right)} \right)^{{ - 1/\left( {m - 1} \right)}} }}.$$

Similarly, let $\partial L/\partial v_{i} = 0$; that is,

18 $$\frac{\partial L}{{\partial v_{i} }} = \mathop \sum \limits_{k = 1}^{N} u_{ik}^{m} \left( {\mathop \sum \limits_{{r \in {\Omega }_{k} }} K\left( {\left( {b_{k} x_{k} - b_{k}^{2} v_{i} } \right) + \beta_{k} \left( {b_{k} \overline{x}_{k} - b_{k}^{2} v_{i} } \right)} \right)} \right) = 0$$

From (11), we obtain

19 $$v_{i} = \frac{{\mathop \sum \nolimits_{k = 1}^{N} \mathop \sum \nolimits_{{r{\Omega }_{k} }} Kb_{k} \left( {x_{k} + \beta_{k} \overline{x}_{k} } \right)u_{ik}^{m} }}{{\mathop \sum \nolimits_{k = 1}^{N} \mathop \sum \nolimits_{{r \in {\Omega }_{k} }} Kb_{k}^{2} \left( {1 + \beta_{k} } \right)u_{ik}^{m} }}.$$

**Figure 4:**
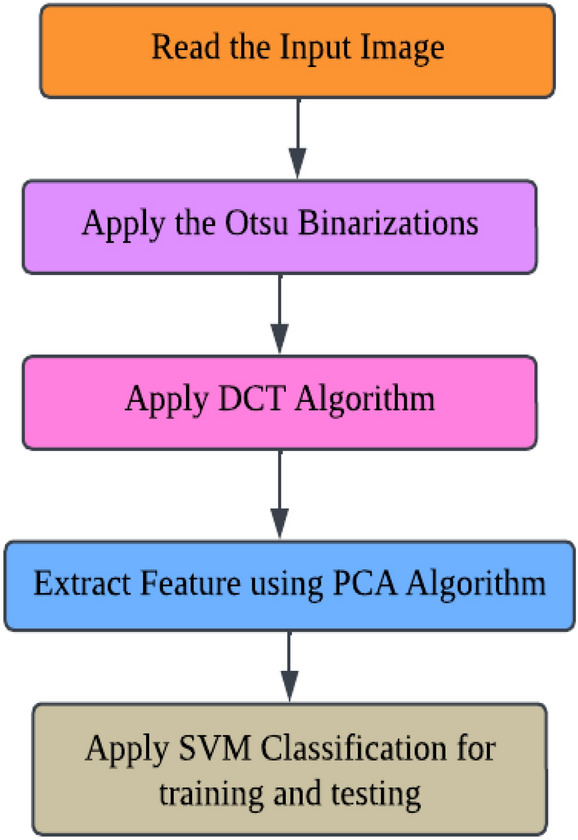
Flowchart of proposed work with DWT, PCA and SVM classification.

The Algorithm determines which pixel goes below the foreground and background. A picture or image with an abundant grey level is regenerated into less grey-level pictures, and comparisons are completed on each pixel intensity with a reference worth (Threshold). If the input picture f (x, y) and the Binary version is g (x, y), then g (x, y) = 1 if f (x, y) >  = T or 0 otherwise. PCA has been used to shrink the number of surplus characteristics from the data set. The above-given algorithm steps can be understood using the below-given block diagram, Fig. 4.

## Implementation

The given section describes the system's hardware and software requirements; the functions and methods consumed in the proposed work are discussed in detail. We used the MATLAB simulator for the implementation.

### Material and methods

**Figure 5:**
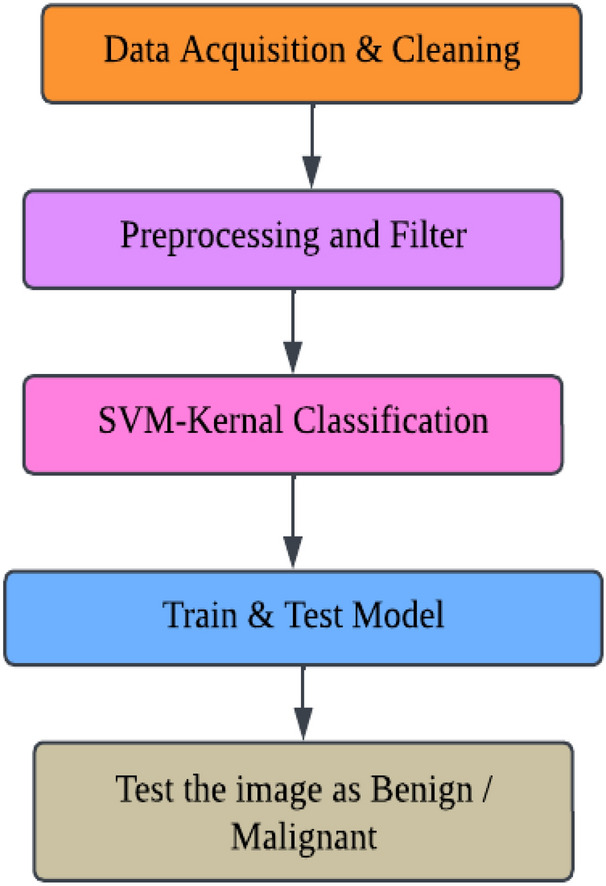
Dividing the training and testing of data.

This research uses the input magnetic resonance imaging brain image to implement the Algorithm. Instead of a different brain scan, an MRI picture provides comprehensive information about the brain tissues. Initially, the brain reflection is cheered whether it is pretentious by the tumor. So, if the tumor constituency is extracted from the brain picture, the number of pixels in the tumor section is computed^39^. If the number of pixels is zero, the classification displays the brain image without a tumor region. Otherwise, the image demonstrates the brain image with the tumor region. If the brain image has a tumor province, this image must do the next stepladder. The first step is to check if the brain image is affected by a tumor. If it is, the tumor region is extracted from the brain image and the number of pixels in the tumor region is computed. If there are no pixels in the tumor region, the algorithm classifies the brain image as without a tumor. Otherwise, the image is classified as having a tumor region. After identifying the tumor region in the brain image, the next step is to use an SVM (Support Vector Machine) classifier to train and test the data. SVM is a popular machine learning algorithm used for classification, regression, and other tasks. Figure 5 shows that we train and test the data through SVM Classifier. If overfitting occurs then regularization techniques such as L1 and L2 regularization can be used to add a penalty to the loss function of the model, which can reduce overfitting by discouraging the model from learning complex, unnecessary patterns in the training data.

In the next step, the filter is employed to eliminate the noise from the brain image; to detach the noise, many filters can be used. MRI does not adulterate heaps of noise. Thus, the average filter eliminates the brain image noise during this system. The regularized image is obtained once preprocessed and can be used in the next step. The normalized image is used to handle quickly in the next step. The SVM classifier uses input data from the feature set and is classified into two achievable categories. The high dimensional feature of the non-linear SVM work in a very significant way. Kernel functions are used to enhance the margin of classification. It is simple to use in sensible image processing and provides a clear understanding of the classification. To realize the segmented picture in this work that is being offered, techniques such as RBF, linear, and polygonal are used. Several data set properties have been evaluated and analyzed to develop a classification system, including Energy, Homogeneity, Correlation, Mean, Entropy, Standard Deviation, RMS, Variance, Smoothness, Skewness, Kurtosis, IDM, and Contrast, amongst others.

**Figure 6:**
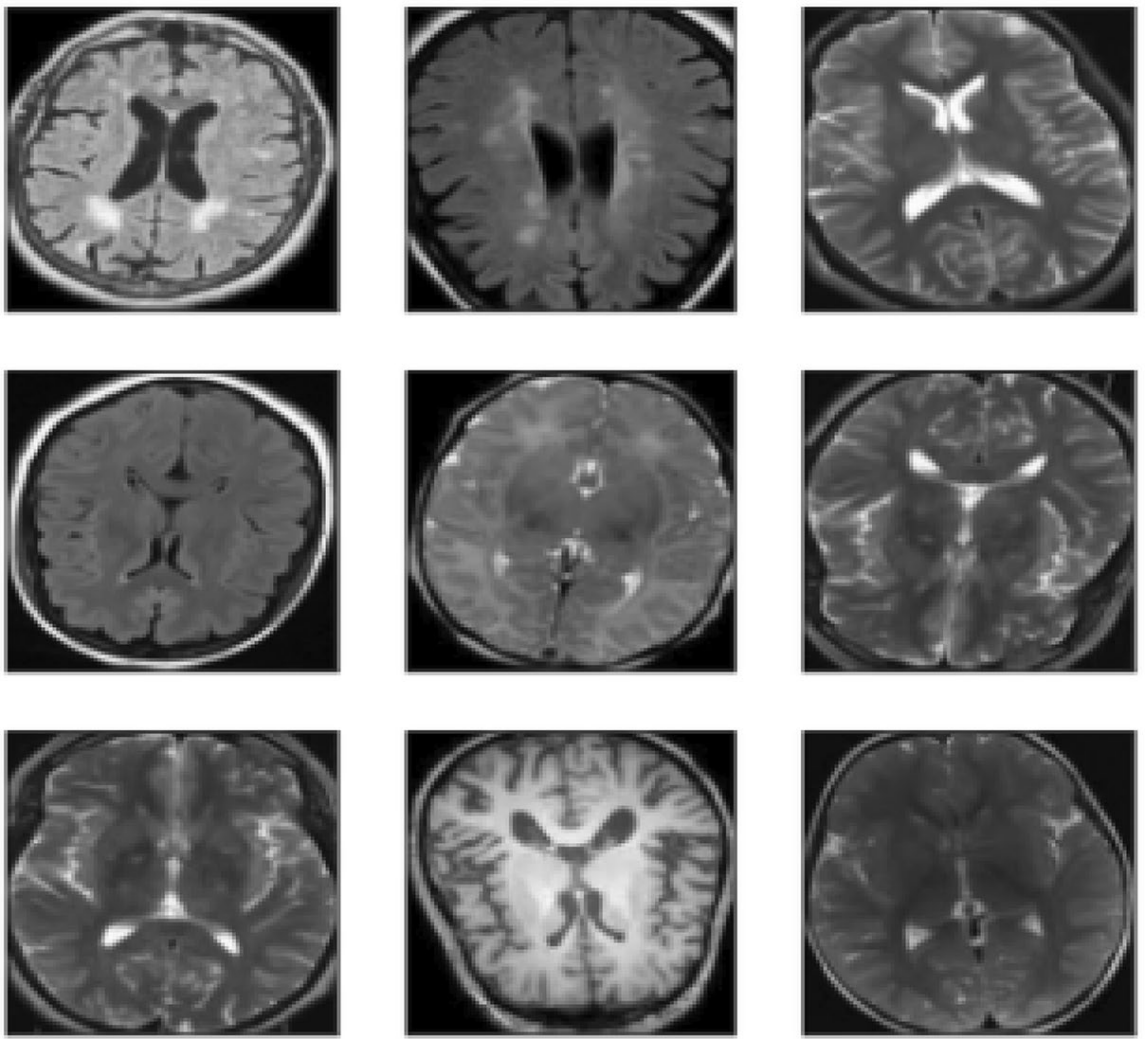
Classification of brain tumor dataset.

**Figure 7:**
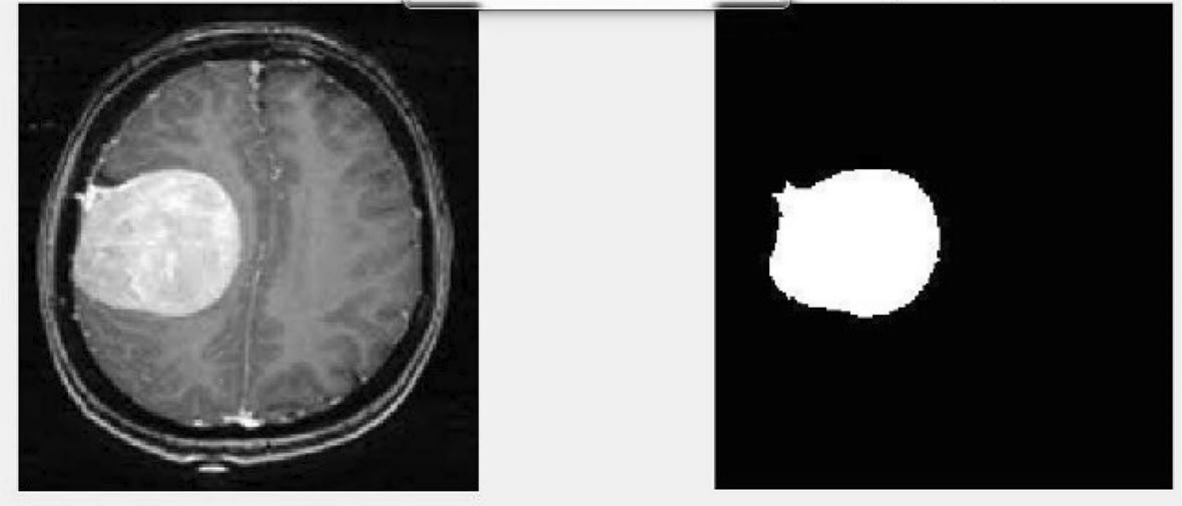
Brain tumor detection screen of benign tumor.

**Figure 8:**
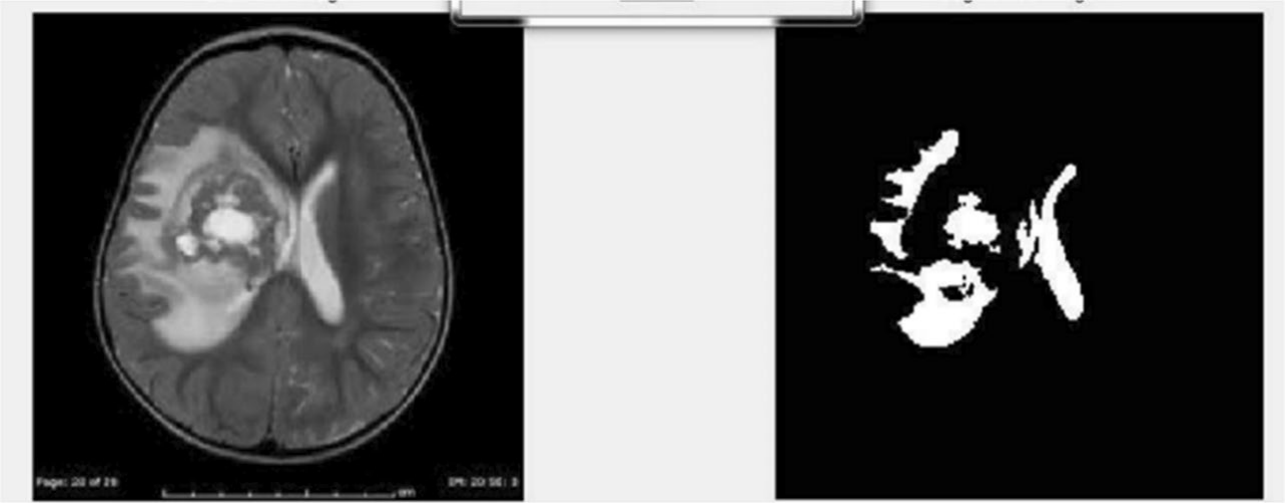
Brain tumor detection screen of malignant tumor.

**Figure 9:**
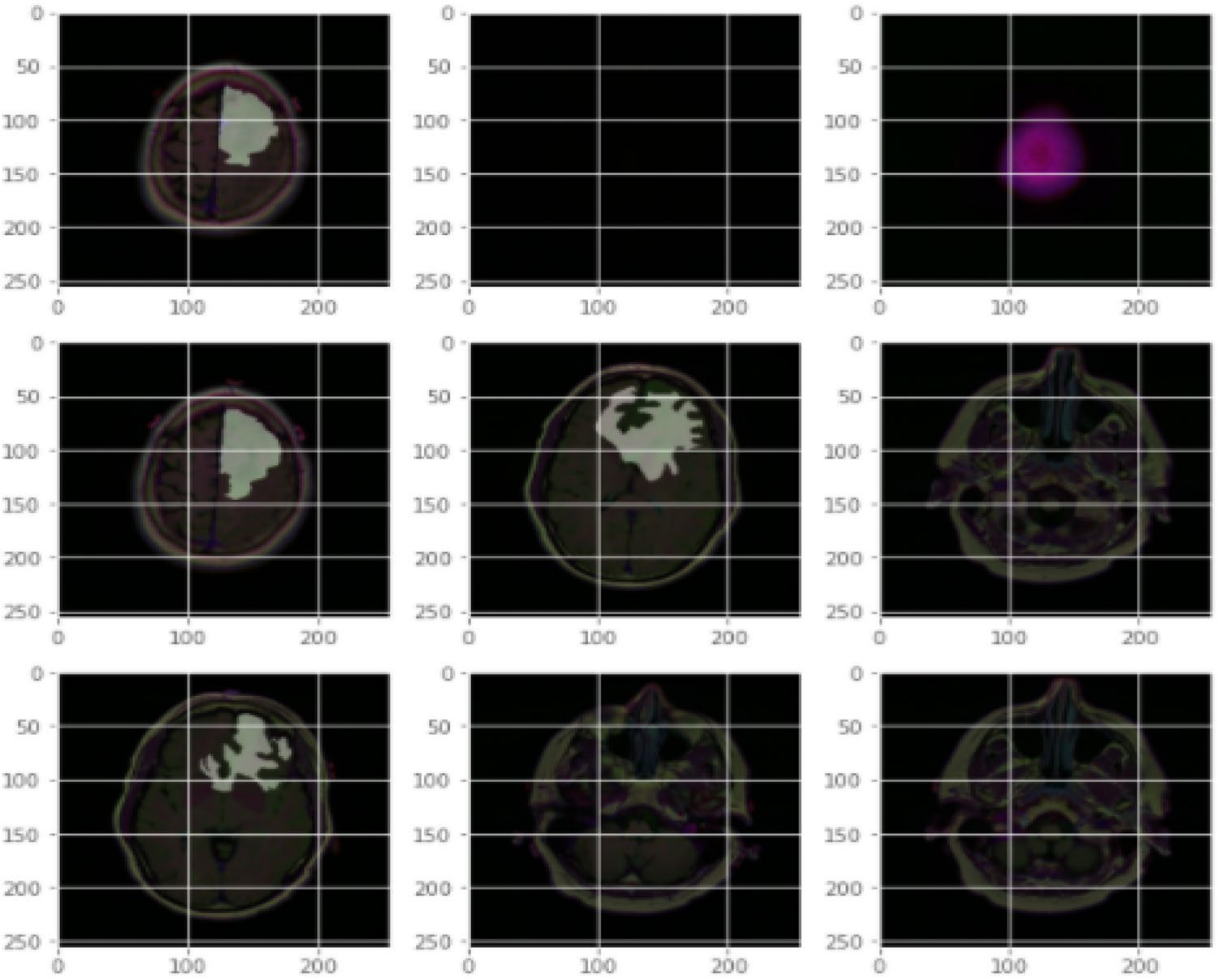
Segmentation and classification of brain tumor detection.

Figure 6 describes the data set, and the implemented method is demonstrated in Figs. 7 and 8. In Fig. 9, first, an image can be selected by the Load MRI Image Button.

Otsu's binarization method for image segmentation and then using a button named Segmented image creates the segmented image. It calculates texture-based features like mean, entropy, root mean square, variance, standard deviation, skewness, smoothness, kurtosis, IDM, etc., with the help of GLCM and extracts the features using PCA. Through accuracy features, we can calculate SVM with various They then extracted super pixels and PCA-based features to improve segmentation and enhance the robustness of the algorithm. Different features are exhibited by multi-sequence MRI data depending on collection settings including pulse sequence and contrast processes, suggesting the potential use of the suggested approach. Super pixels and the principal component analysis (PCA) technique were important in obtaining metabolic information for precise classification, allowing them to employ multimodal MRI to detect the varied patterns of glioma tumors. To simulate the difficulty of detecting diverse patterns in brain tumors, we employed the SVM method for segmentation in the simulation, which resulted in the formation of eight clusters. In addition, morphological techniques were employed to isolate the tumor from the background picture. It was determined that the tumor had spread by using segmentation and morphological techniques. The proposed algorithm showed promising results in detecting brain tumors in MR images, and the use of superpixels and PCA-based features improved the accuracy and robustness of the algorithm. The proposed scheme also has the potential for detecting brain tumors in multi-sequence MRI data kernel functions.

### Result analysis

The presentation of the projected system is evaluated in this document fragment. Based on experiments, the results are assessed, and their relevant performance is given in this paper.

**Figure. 10:**
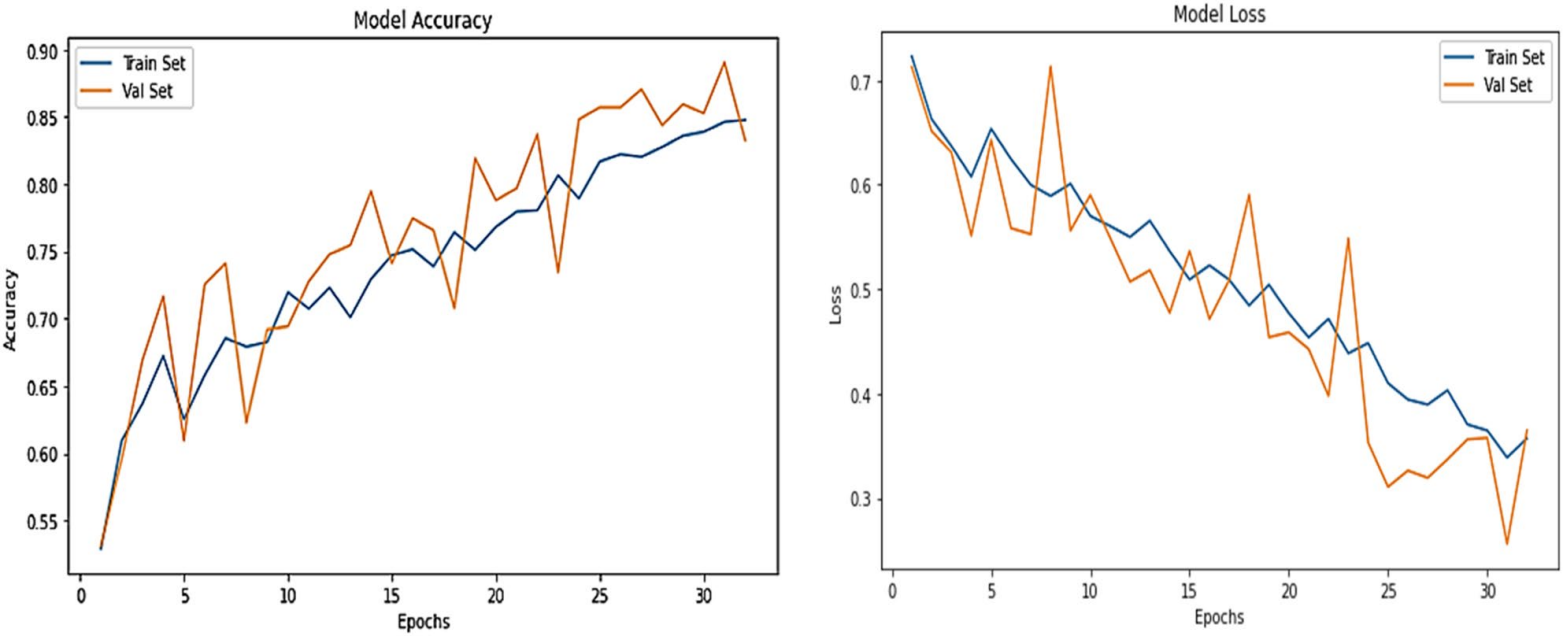
Comparative analysis of accuracy observed for malignant data set.

Figure 10 describes the proposed system’s accuracy for the malignant data set.

**Figure 11:**
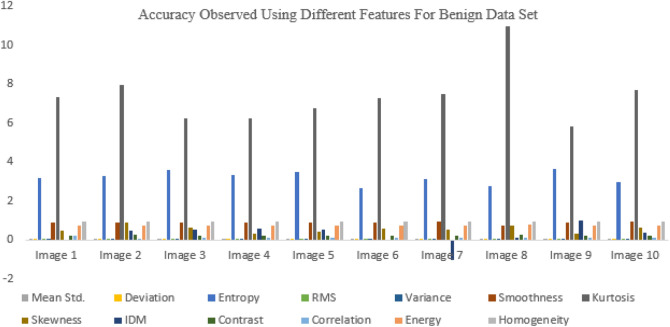
Accuracy observed using different features for benign data set.

**Figure 12:**
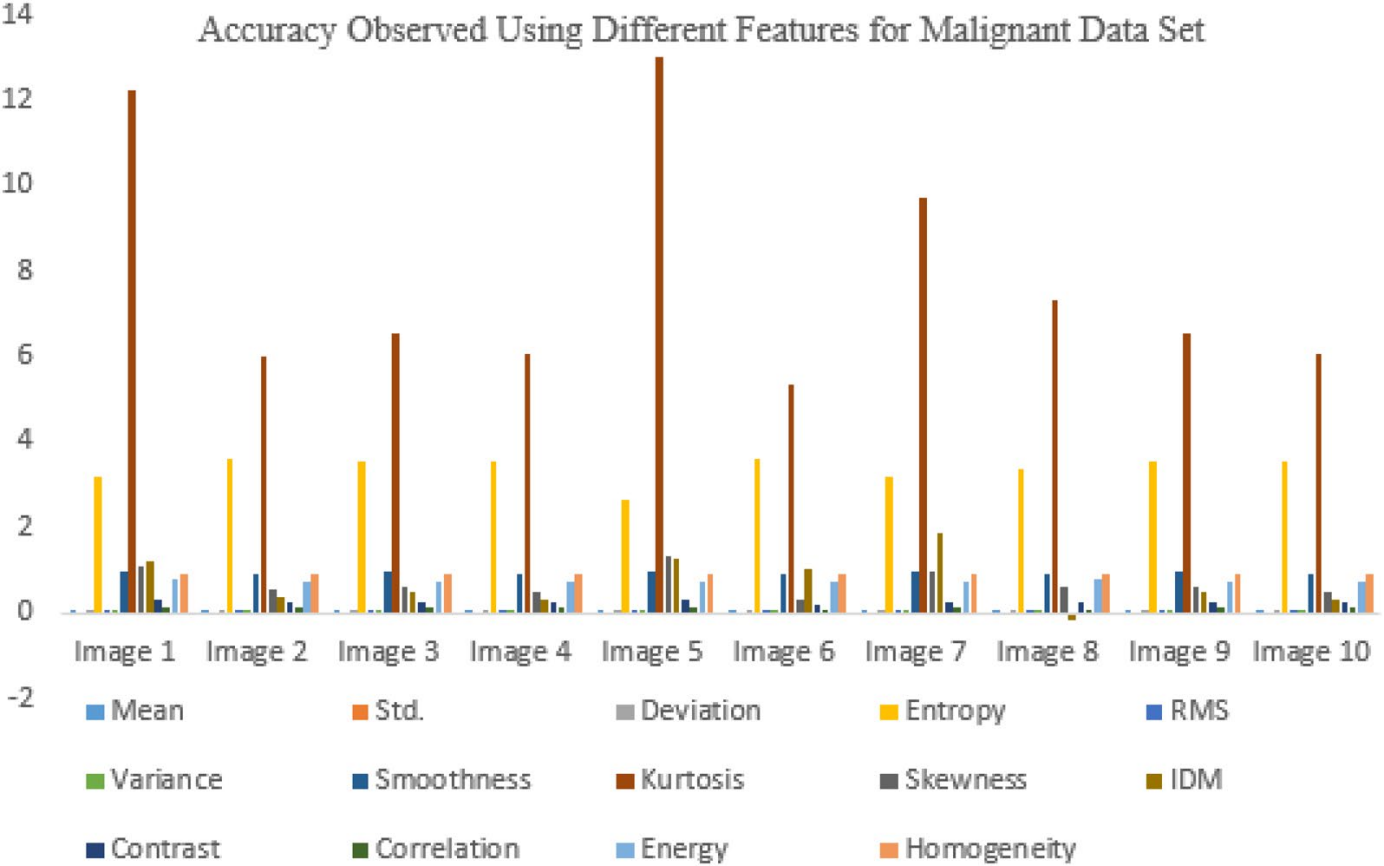
Computation of accuracy observed using different features for malignant data set.

In Figs. 11, 12, the malignant data set and the calculations are performed on it for different features.

### Kernel functions

An uphill task awaits you if you are tasked with selecting an appropriate classification method for the picture. As a result, the non-linear support vector machine^39^ performs very well on high-dimensional distinct sets.

**Figure 13:**
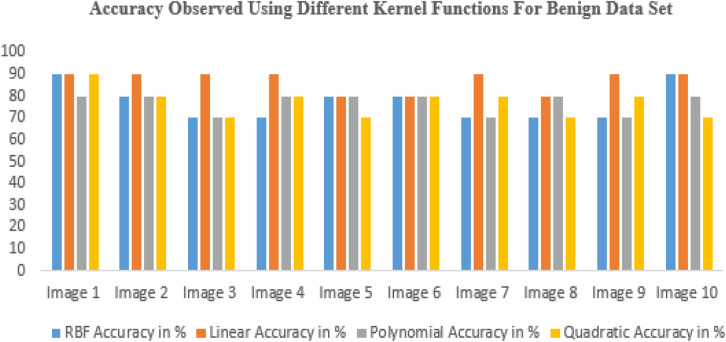
Accuracy observed using different kernel functions for benign data set.

In this study, the segmented picture is located using a few different kernel function approaches, such as RBF, linear, and polygonal^40^. Figure 13 shows the accuracy results for other kernel functions for benign data sets, proving that the linear accuracy method results well. Median filtering is used to prepare input photos from the database that are representative of brain tumors. Extraction of features based on PCA and superpixels aided in the segmentation process. The suggested approach will also be useful for multi-sequence MRI data, which varies in quality depending on factors like the pulse sequence and contrast mechanisms used during acquisition. Glioma tumors, with their varying appearance from patient to patient, are best detected by multimodal magnetic resonance imaging (MRI). Super pixels and the principal component analysis (PCA) method play crucial roles in obtaining metabolic information that enables precise categorization from MRI data. Therefore, our suggested technique is capable of detecting brain tumors with diverse patterns with relative ease. The SVM technique was employed for segmentation and successfully isolated the tumor region from the rest of the picture in the simulation. In addition, the morphological procedure isolates the tumor from the background picture. Tumor damage is identified using segmentation and morphological techniques. There are different types of uncertainties associated with models, such as aleatoric and epistemic uncertainties. Aleatoric uncertainties are related to the inherent randomness in the data, while epistemic uncertainties are related to the limitations of the model and the data used to train it. Both types of uncertainties should be reported to provide a complete picture of the model's reliability and validity. Reporting uncertainties can also help identify areas for improvement in the model, such as the need for more diverse training data, improved model architecture, or additional features. It can also aid in better understanding the limitations of the model and how it can be appropriately used.

**Figure 14:**
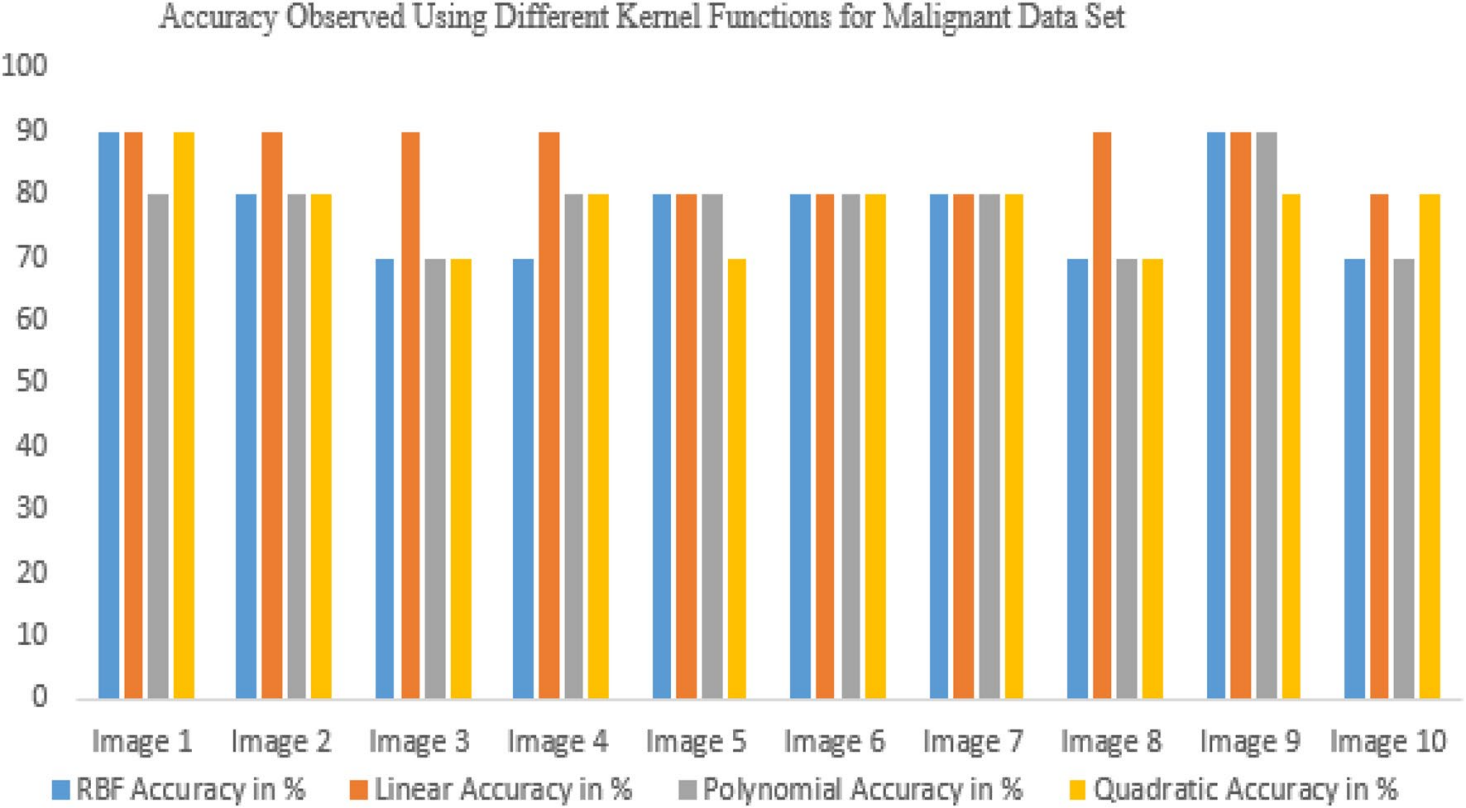
Accuracy observed using different kernel functions for malignant data set.

Figure 14, shows the accuracy results for different kernel functions^28,29^ for the malignant data set, proving that the linear accuracy method results well.

Table 2 describes a comparison of different classifiers based on their performance metrics such as accuracy, TP, TN, FP, FN, and ROC. It is important to note that the performance of a classifier can be affected by various factors such as the quality and size of the dataset, the choice of features used for classification, and the hyperparameters selected during training.

**Table 2:**
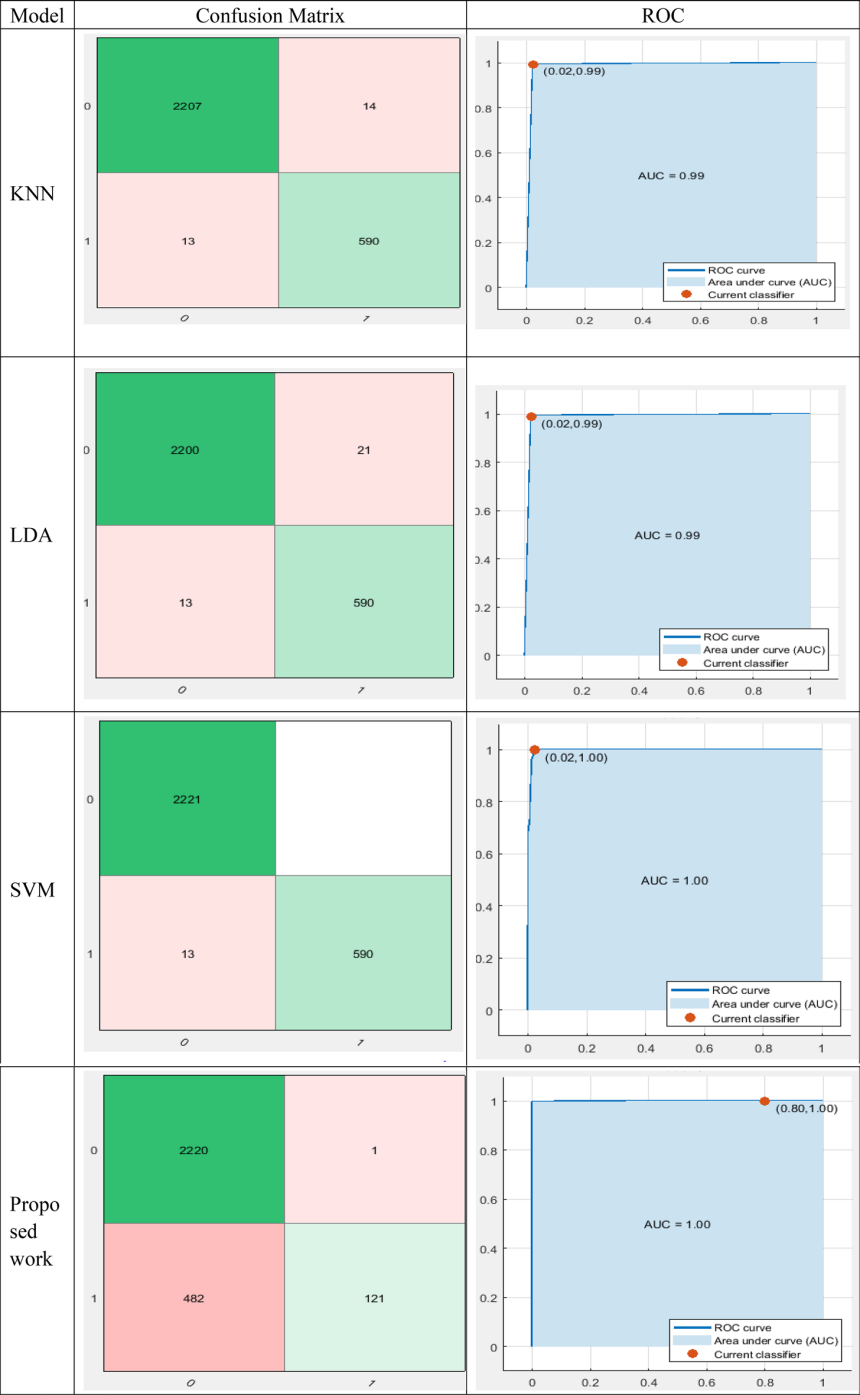
Confusion matrix and ROC (Without Using PCA).

As a dimensionality reduction method, principal component analysis (PCA) can help classifiers perform better by lowering the number of features they need to make a decision. When working with high-dimensional datasets, where the number of features is substantially more than the number of samples, this can be quite helpful. Using the use of principal components analysis (PCA), the original collection of characteristics may be reduced to a more manageable subset of linearly uncorrelated features. The variation explained by each of these principal components is listed from highest to lowest; the first component explains the most variance in the data, and so on. By eliminating extraneous features and simplifying the classification issue, principal component analysis (PCA) has the potential to boost classifier performance. However, it is important to note that the performance gain obtained by PCA can depend on the specific dataset and classifier being used and that in some cases, PCA may not be necessary or may even lead to a decrease in performance. The performance of classifiers can be compared based on various metrics such as accuracy and confusion matrices, the use of PCA can also play a role in improving classifier performance by reducing the dimensionality of the feature space show in Table 3.

**Table 3:**
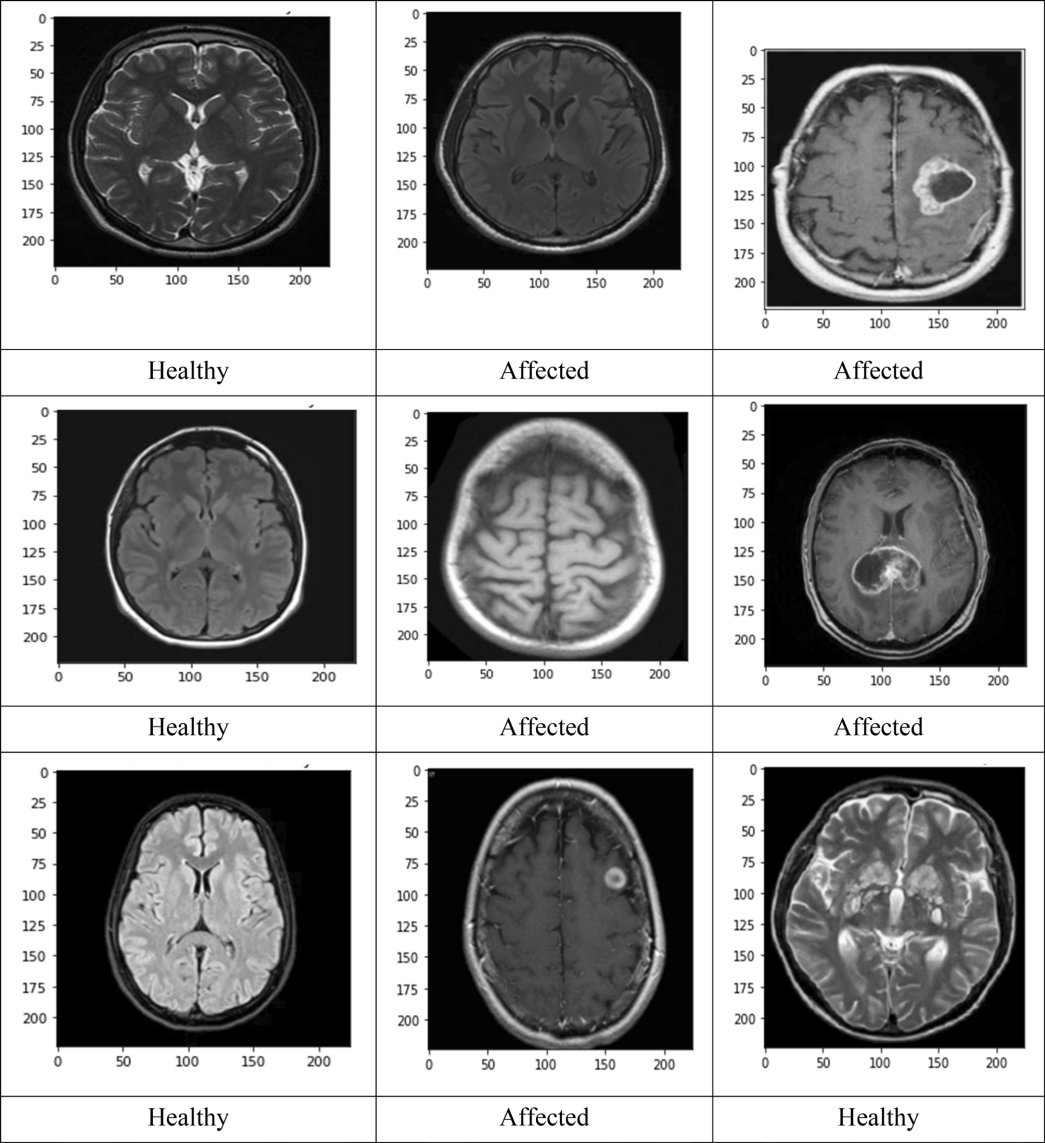
Result of Proposed method on a different image and their prediction about the disease.

Table 4 shows the Otsu's method, level set, watershed, K-means, discrete wavelet transform, and convolutional neural networks all have a reaction time of 12.51 s, 9.217 s, 7.267 s, 4.574 s, 2.677 s, and 2.516 s, respectively. Otsu’s approach takes the longest since it only analyses the image once and involves calculations of irrelevant parts. The suggested work can provide several benefits by combining various methods. May segment pictures to boost SVM classification accuracy and significantly minimize feature space size. Face identification, texture categorization, and object recognition are just a few of the areas where this method has been demonstrated. proposed work may have various advantages over conventional approaches like manual feature extraction and categorization^46^. To begin with, it is a data-driven method that eliminates the need for human feature engineering by automatically extracting the most important characteristics from the data. Secondly, it can handle high-dimensional data, which is often difficult for traditional methods. Thirdly, it can improve classification accuracy by reducing the noise and redundancy in the feature space and by segmenting the images to improve the SVM classifier's performance. However, it is important to note that the effectiveness of the proposed work can offer several advantages can depend on the specific dataset and classification task being used. Additionally, this approach can require significant computational resources, particularly when dealing with large datasets or complex feature spaces. Therefore, it is important to carefully evaluate the advantages and limitations of the proposed work can offer several advantages and other classification approaches when selecting the best method for a given task. However, with the level-set process, the image is split ahead of time. K-means takes the average of each image and uses that to similar group photos together. The rules upon which the watershed technique is built include the use of morphological operators, the filtering of regions, and the identification of the exact position of the outline^47^. The DWT technique simplifies a huge image by separating it into low and high-frequency bands with four sub-bands: LL, LH, HL, and HH. In terms of performance metrics and reaction time, the proposed method outperformed competing algorithms when applied to the problem of brain tumours.

**Table 4:** Comparative analysis of recall, precision, F-measure, and accuracy of the proposed method with existing methods.

Authors	Method	Recall	Precision	F-measure	Accuracy
^41^	Otsu’s	0.681	0.892	0.773	0.714
^42^	watershed algorithm	0.782	0.827	0.804	0.782
^43^	level set method	0.775	0.863	0.817	0.804
^44^	K-means algorithm	0.931	0.764	0.839	0.843
^45^	DWT algorithm	0.885	0.867	0.876	0.869
Proposed work	Optimum global thresholding	0.869	0.952	0.909	0.987

## Conclusion and future work

Data Analytics is now the norm for gathering insights from the internet and large databases. Users frequently submit queries to obtain results tailored to their specific data needs. Techniques for extracting images and other features are taught in this research. Issues with medical image search and the factors that allow for precise database image separation are discussed. Findings from this study confirmed that primary features are calculated during the content-based image retrieval procedure. However, two of them still aren’t helpful because of how similarly colored and textured the images in a medical database are. Kernel functions are used to broaden the boundary of the classification. Several kernels, including linear ones, polynomial ones, radial basis function (RBF) ones, etc., are used in SVM. Kernel SVM is straightforward to implement in practical image processing and offers insightful classification clarity. This method uses its techniques to locate the segmented image, such as RBF, linear, and polygonal. Classification strategies have considered a wide variety of data set characteristics, including RMS, Entropy, Variance, Mean, Standard Deviation, Smoothness, Contrast, Correlation, Energy, Homogeneity, etc. When applying Otsu's segmentation to the Brain MRI dataset, excellent results are obtained, and PCA achieves better results when reducing features. Specifically, we have analyzed specific brain MRI data to compare four different kernel functions of SVM. In the long run, we'll be able to use other feature reduction methods and reach them to grasp the accuracy finally. In addition, the scientist can use a variety of hybridized learning algorithms by plugging them into a specific slot in the dataset. This study's research focuses on segmenting and classifying various brain MRI images using machine learning and SVM. Limitation of this research is the performance of the image segmentation and classification techniques outlined in this research is influenced by the inherent variability in medical images, particularly in brain MRI scans. Variations in image resolution, contrast, and artifacts may impact the accuracy of the results.

## Data Availability

The datasets are available https://www.kaggle.com/datasets/navoneel/brain-mri-images-for-brain-tumor-detectionand. Source code used for study are available from the corresponding author on reasonable request.
